# The effect of combined application of Streptomyces rubrogriseus HDZ-9-47 with soil biofumigation on soil microbial and nematode communities

**DOI:** 10.1038/s41598-019-52941-9

**Published:** 2019-11-15

**Authors:** Na Jin, Xiuliang Lu, Xueyan Wang, Qian Liu, Deliang Peng, Heng Jian

**Affiliations:** 1 Key Laboratory of Pest Monitoring and Green Management, Ministry of Agriculture, Department of Plant Pathology, China Agricultural University, Beijing, 100193 China; 2 State Key Laboratory for Biology of Plant Diseases and Insect Pests, Institute of Plant Protection, Chinese Academy of Agricultural Sciences, Beijing, 100193 China; 3 Department of Horticulture, Beijing Vocational College of Agriculture, Beijing, 102442 China

**Keywords:** Agroecology, Parasite host response

## Abstract

*Meloidogyne incognita* causes significant damage to many different crops. Previous studies showed that *Streptomyces rubrogriseus* HDZ-9-47 is a promising biocontrol agent. Combining it with biofumigation improved its efficacy against *M*. *incognita*. In the present study, the reason for the improved efficacy of the combination was investigated by analyzing its impact on both the soil microbial and the nematode communities in the field. The results showed that the combined application reduced root galls by 41% and its control efficacy was greater than each treatment alone. Cultivation-based analyses showed that the combination treatment affected the soil microbial community. Actinomycetes and bacterial densities were negatively correlated with the root knot score. In contrast, the fungal densities were positively correlated with the root knot score. Denaturing gradient gel electrophoresis (DGGE) results showed that the combination of *S. rubrogriseus* HDZ-9-47 and biofumigation enriched beneficial microbes and reduced certain soil-borne fungal phytopathogens, thereby enhancing the efficacies of both *S. rubrogriseus* HDZ-9-47 and biofumigation against *M. incognita*. And HDZ-9-47 could colonize in soil. The total abundance of nematode and plant parasites, the ratio of soil fungivore nematode to fungivore plus bacterivore nematode, and the nematode diversity indices all decreased with the combination treatment. Overall, the results of this study demonstrate that combined application of HDZ-9-47 with biofumigation was a useful and effective approach to suppress *M. incognita* by manipulating soil microbial communities in field.

## Introduction

*Meloidogyne incognita* is a serious agricultural pest. It attacks a wide range of crops and causes dramatic yield losses^1^. For decades, *M. incognita* was controlled mainly with chemical nematicides. However, most of these products have been banned because of their toxicity to soil ecosystems and human health. Therefore, safe, alternative control methods to *M. incognita* are urgently required. Biocontrol is an attractive nematode management strategy^2^.

Microorganisms are rich natural sources of nematode biocontrol agents. *Streptomyces* play important roles in controlling plant parasitic nematodes^3,4^. Rashad *et al*. isolated 112 *Streptomycetes* from 20 marine samples. Twenty-eight strains exhibited nematicidal activity *in vitro* and under greenhouse conditions^3^. Until now, however, only a few biocontrol agents have ever been commercialized and applied on a large scale in the field^5^. A crucial limiting factor of the successful field application of biocontrol agents is their inconsistent performance under various environmental conditions^6^. However, applying biocontrol agents in combination with soil biofumigation may solve this problem^7^. Li *et al*. reported that combining soil biofumigation with antagonistic *Pseudomonas reinekei* SN21 was highly effective against *M. incognita* in the field^8^. Biofumigation applied as soil incorporation of cabbage residues with subsequent water saturation of the soil and coverage with plastic foil, in combination with the biocontrol strain *Streptomyces rubrogriseus* HDZ-9-47 was more effective against *M. incognita* than the nematicide fosthiazate in a field trial^9^. However, the reason of improved efficacy of the combination of biocontrol agents with biofumigation against *M. incognita* are poorly known.

Once its materials are incorporated into the soil, biofumigation enriches soil carbon and nitrogen and may alter soil microbial and nematode community structure^10–12^. Biocontrol agents may also affect indigenous microbial and nematode community composition^13^. Soil microbes and nematodes usually play important roles in agro-ecosystems. They influence soil nutrient cycling, organic matter formation and decomposition, soil structure, and plant systemic resistance. They may also suppress plant pathogens^14^. Biocontrol agents utilize the carbon and nitrogen provided by biofumigation, thereby increasing their viability and competitiveness in the soil^14^. The application of antagonistic *Bacillus amyloliquefaciens* strain BS211 with biofumigation affected certain microbial densities, increased soil bacterial diversity, and suppressed *Phytophthora* blight in pepper^15^. Valdes *et al*. found that soil biofumigation decreased plant parasitic nematode densities while increasing those of bacterivorous nematodes^16^. So, analyzing the effects of combined application of biocontrol agents with biofumigation on the soil microbial and the nematode communities is helpful for understanding the mechanism of improved performance in field.

The potential environmental risks of introducing biocontrol agents have seldom been studied^17^. The introduction of biocontrol agents may perturb indigenous microbial densities^13^. Before deploying the combination of biocontrol agents and biofumigation on a large scale, it is necessary to assess their impact on soil microbial communities.

*Streptomyces rubrogriseus* HDZ-9-47 was isolated from the eggs of *Meloidogyne* sp.^18^. Our earlier study showed that HDZ-9-47 together with biofumigation reduced *M. incognita* in the field^9^. In the present study, the effects of the combined application of HDZ-9-47 with biofumigation on soil microbial and nematode communities were investigated. The results will help to improve the field efficiency of biocontrol agent against *M. incognita*.

## Results

### *S. rubrogriseus* HDZ-9-47 combined with soil biofumigation improves control efficacy against *M. incognita*

Efficacies of the various treatments against *M. incognita* are showed in Table 1. The root gall scores in the biofumigation and combination treatments were significantly lower than that of the untreated control at 90 d after transplanting (*P* < 0.05, Table 1). Therefore, combination of HDZ-9-47 with biofumigation could control *M. incognita* in the field.

**Table 1:** Effect of the combination of HDZ-9-47 and soil biofumigation on *Meloidogyne incognita* control at 90 d after transplanting under field conditions in autumn 2014.

Treatment	90 d	
	^e^Root gall score	Root gall reduction
^a^C-H	4.47 ± 1.50 a	41% a
^b^H	7.00 ± 0.53 b	8% b
^c^C	5.33 ± 1.21 a	30% a
^d^CK	7.60 ± 0.35 b	—

^a^Combination treatment of *Streptomyces rubrogriseus* HDZ-9-47 and soil biofumigation with cabbage. ^b^HDZ-9-47. ^c^Soil biofumigation with cabbage. ^d^Equal volume of water without nematicide or biocontrol agent. ^e^Root gall was assessed using a 0–10 rating scale according to Bridge and Page (1980). Data are means ± SD for 15 replications. Means followed by different letters in the same columns are significantly different from each other at the 0.05 probability level according to Tukey’s test.

### Effect of the combination of HDZ-9-47 and biofumigation on the soil microbial community

**Figure 1:**
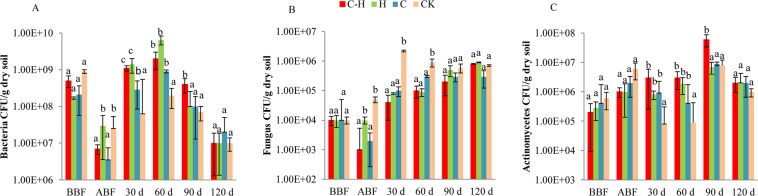
Effect of combination of HDZ-9-47 and biofumigation on the densities of soil culturable bacteria (**A**), fungi (**B**), and actinomycetes (**C**) at different sampling times. C-H: combined applications of *Streptomyces rubrogriseus* HDZ-9-47 and cabbage biofumigation. H: application of HDZ-9-47 alone. C: soil biofumigation with cabbage. BBF and ABF: before- and after biofumigation, respectively. Error bars represent standard deviation. The different letters on each bar within same sampling time represent significant differences at the 0.05 level based on Tukey’s multiple comparison test.

The cultivable method analysis showed that the bacterial, fungal, and actinomycetes densities did not significantly differ among treatments before biofumigation (Fig. 1).

As showed in Fig. 1A, the culturable bacterial densities in the combined application of HDZ-9-47 with biofumigation treatment and biofumigation alone treatment were significantly lower than that in the control treatment after biofumigation (*P* < 0.05). At 30 and 60 d post transplantation, the culturable bacterial densities in the application of HDZ-9-47 or biofumigation alone or combination treatments were significantly higher than that in the control treatment, and those in the application HDZ-9-47 alone and combined with biofumigation treatments were significantly higher than that in the biofumigation alone treatment (*P* < 0.05). At 90 d post transplantation, the culturable bacterial density in the combination treatment was significantly lower than other treatments (*P* < 0.05). At 120 d post transplantation, the culturable bacterial density in each treatment showed no significantly difference (*P* > 0.05). The results indicated that soil biofumigation decreased the culturable bacterial densities, and application of HDZ-9-47 alone or combined with biofumigation increased the culturable bacterial densities at the early stage of tomato growth.

After biofumigation, the culturable fungal density in the combined application of HDZ-9-47 with biofumigation treatment or biofumigation alone treatment were significantly lower than that of the control, respectively (*P* < 0.05, Fig. 1B). At 30 and 60 d post transplantation, the culturable fungal densities in the application of HDZ-9-47 or biofumigation alone or combination treatments were significantly lower than that of the control, respectively (*P* < 0.05, Fig. 1B). At 90 and 120 d post transplantation, the culturable fungal density showed no significantly difference among treatments (*P* > 0.05, Fig. 1B). The results indicated that soil biofumigation and application of HDZ-9-47 decreased the culturable fungal densities in the early stage of tomato growth.

Actinomycetes densities did not significantly differ among treatments after biofumigation (*P* > 0.05, Fig. 1C). At 30 and 60 d post transplantation, the culturable actinomycetes density was significantly higher than that of the control (*P* < 0.05, Fig. 1B). At 90 d post transplantation, the culturable actinomycetes density in the combination treatment was significantly higher than other treatments (*P* < 0.05, Fig. 1B). At 120 d post transplantation, the culturable actinomycetes density showed no significantly difference among treatments (*P* > 0.05, Fig. 1B). The results indicated that soil biofumigation and application of HDZ-9-47 increased the culturable actinomycetes densities in the early stage of tomato growth.

Correlation analysis showed that the culturable bacterial density was negatively correlated with the root knot score at 90 d after transplanting (*r* = −0.445*; *P* < 0.05), and the culturable fungal density was significantly positively correlated with the root knot score at 90 d after transplanting (*r* = 0.535*; *P* < 0.05).

**Figure 2:**
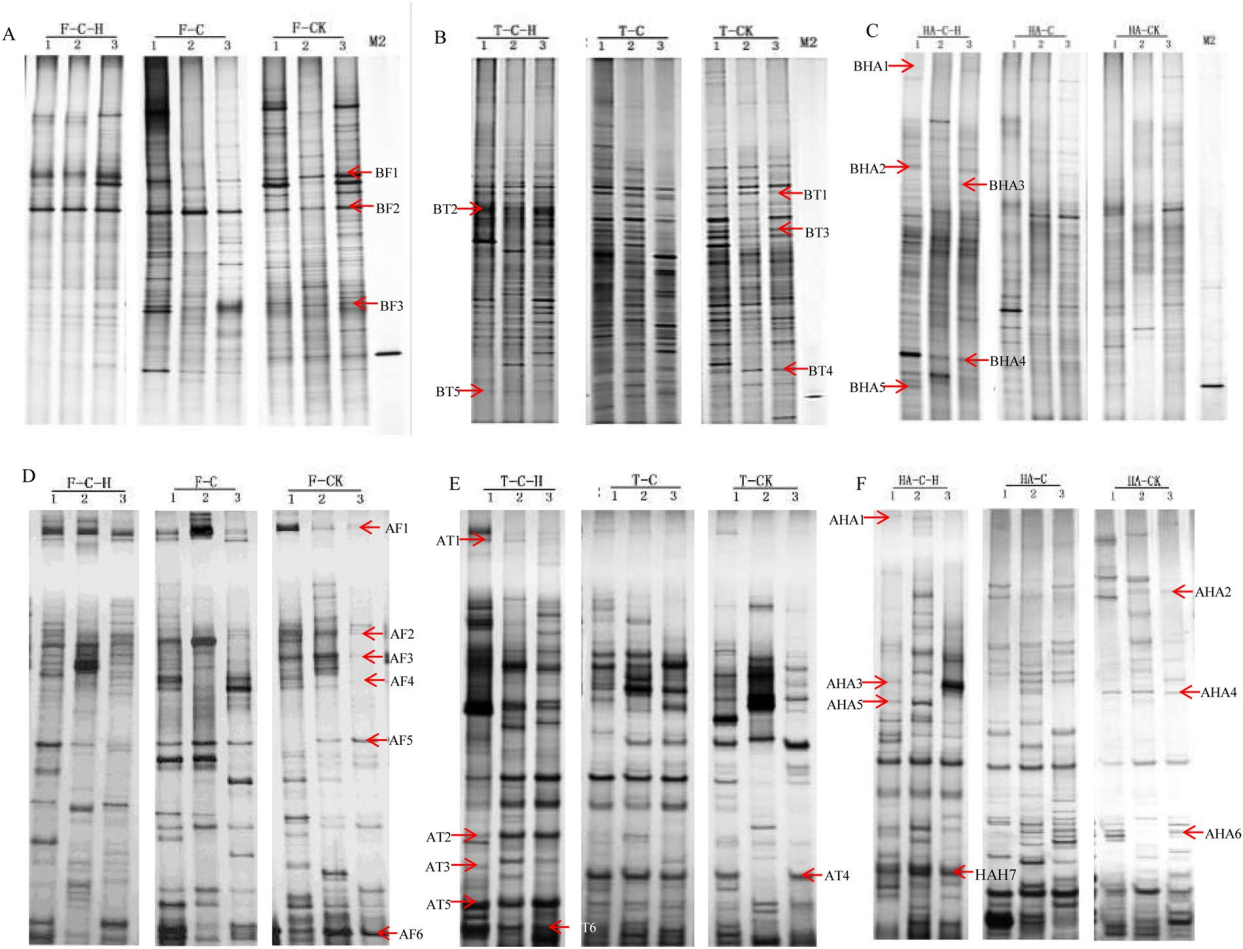
DGGE patterns of bacterial 16 S rDNA genes (**A–C**) and fungal ITS (**D–F**) in different treatments immediately after biofumigation (**A,D**), and at 30 d (**B,E**) and 120 d (**C,F**) after transplanting. C-H: combination of *Streptomyces rubrogriseus* HDZ-9-47 and biofumigation by cabbage. H: application of HDZ-9-47 alone. C: soil biofumigation with cabbage. M2: marker of a PCR product obtained from HDZ-9-47. F, T, and HA: samples collected before biofumigation and at 30 d and 120 d after transplanting, respectively. All images in this figure were partly selected from original images of DGGE assays on bacteria and fungi and spliced together.

The effects of all treatments on the soil bacterial and fungal communities were further analyzed by PCR-DGGE using three replications per treatment. Certain bands were common to all treatments after biofumigation (Fig. 2A,D). These included the bacterial bands BF1, BF2, BF3 and the fungal bands AF1, AF2, AF3, AF4, AF5, and AF6 (Fig. 2A,D, Tables 2 and 3). Therefore, these soil bacteria and fungus were stable and unaffected by biofumigation.

**Table 2:** Identification of selected bacterial 16S rDNA sequences and their GenBank accession numbers.

Band Number	Accession number	Similar strain	Classification
Band BF1	MK277303	uncultured *Planomicrobium* sp.	Firmicutes
Band BF2	MK277304	Oxalobacteraceae bacterium	Proteobacteria
Band BF3	MK277303	uncultured gamma proteobacterium	Proteobacteria
Band BT1	MK277310	*Oxalobacteraceae* bacterium	Proteobacteria
Band BT2	MK277311	uncultured bacterium	Bacteria; environmental samples
Band BT3	MK277312	uncultured *Fictibacillus* sp.	Firmicutes
Band BT4	MK277313	uncultured *Sphingomonas* sp.	Proteobacteria
Band BT5	MK277314	uncultured Actinomycetales bacterium	Actinobacteria
Band BHA1	MK277305	uncultured *Escherichia* sp.	Proteobacteria
Band BHA2	MK277306	uncultured *Shigella* sp.	Proteobacteria
Band BHA3	MK277307	*Paenisporosarcina* sp.	Firmicutes
Band BHA4	MK277308	*Streptomyces* sp.	Actinobacteria
Band BHA5	MK277309	uncultured Actinomycetales bacterium	Actinobacteria

**Table 3:** Identification of selected fungal ITS sequences and their GenBank accession numbers.

Band Number	Accession number	Similar strain	Classification
Band AF1	MK294736	uncultured Ascomycota	Ascomycota
Band AF2	MK294737	*Thielavia* sp.	Ascomycota
Band AF3	MK294738	uncultured Pseudeurotium	Ascomycota
Band AF4	MK294739	*Pleosporales* sp.	Ascomycota
Band AF5	MK294740	uncultured *Thielavia*	Ascomycota
Band AF6	MK294741	*Pyronemataceae* sp.	Ascomycota
Band AT1	MK294749	uncultured fungus	Fungi; environmental samples
Band AT2	MK294750	*Cladosporium* sp.	Ascomycota
Band AT3	MK294751	uncultured Basidiomycota	Basidiomycota
Band AT4	MK294752	*Davidiella tassiana*	Ascomycota
Band AT5	MK294753	uncultured Ascomycota	Ascomycota;
Band AT6	MK294754	uncultured fungus	Fungi; environmental samples
Band AHA1	MK294742	*Pyronemataceae* sp.	Ascomycota
Band AHA2	MK294743	uncultured Ascomycota	Ascomycota
Band AHA3	MK294744	*Eurotiales* sp.	Ascomycota
Band AHA4	MK294745	*Geomyces* sp.	Geomyces
Band AHA5	MK294746	*Thielavia* sp.	Ascomycota
Band AHA6	MK294747	uncultured Rhizoctonia	Basidiomycota
Band AHA7	MK294748	uncultured fungus	Fungi; environmental samples

At 30 d after transplanting, the bacterial band BT2 and the fungal bands AT1 and AT3 were found only in the combination treatment. However, the bacterial bands BT1, BT3, and BT4 were absent in this treatment (Fig. 2B). A bacterial band BT5 with the same electrophoretic mobility as the 16 S rRNA gene fragment of HDZ-9-47 was present in both the HDZ-9-47 and combination treatments. Therefore, HDZ-9-47 may have colonized in soil (Fig. 2B).

At 120 d after transplanting, the DGGE bands were similar for all treatments but could still be differentiated by the presence of weak bands. As showed in Fig. 2C,F, the DGGE bands in the combination treatment differed from those of the other treatments. The new bacterial bands BHA1, BHA2, BHA3, BHA4 and the fungal bands AHA1, AHA3, AHA5, AHA7 appeared whereas the fungal bands AHA2, AHA4, and AHA6 vanished in the combination treatment. The bacterial band BHA5 with the same electrophoretic mobility as the 16 S rRNA gene fragment of HDZ-9-47 was present in the HDZ-9-47 and combination treatments. Therefore, HDZ-9-47 remained stable in the soil at 120 d after transplanting (Fig. 2C).

### Effect of the combination of HDZ-9-47 and biofumigation on the soil nematode community

A total of 26 nematode genera belonging to 12 families were detected in the soil samples. These included 9 plant parasite, 12 bacterivore, 3 fungivore, and 2 predator/omnivore genera (Table 4).

**Table 4:** Effects of combined application of HDZ-9-47 and biofumigation on nematode taxa.

Nematode taxa	^a^c-p	^b^C-H	^c^H	^d^C	^e^CK
**Plant parasites**		—	—	—	—
Tylenchida		—	—	—	—
Heteroderidae		—	—	—	—
*Meloidogyne*	3	5,172	12,539	7,173	10,426
*Heterodera*	3	0	5	34	0
Tylenchidae		—	—	—	—
*Psilenchus*	2	2	5	4	1
*Tylenchus*	2	2	19	1	27
*Lelenchus*	2	0	0	0	0
*Filenchus*	2	21	35	14	52
*Helicotylenchus*	3	0	0	0	1
Criconematidae	—	—	—	—	—
*Criconemella*	3	0	0	0	0
Dorylaimida	—	—	—	—	—
Longidoridae	—	—	—	—	—
*Longidorella*	4	0	0	1	1
**Bacterivores**	—	—	—	—	—
Rhabditida	—	—	—	—	—
Cephalobidae	—	—	—	—	—
*Chiloplacus*	2	33	49	69	72
*Acrobeles*	2	0	0	0	3
*Cervidellus*	2	2	11	8	29
*Arcobeloides*	2	2	0	0	1
*Cephalobus*	2	281	127	174	224
*Eucephalobus*	2	2	52	12	54
Rhabditidae	—	—	—	—	—
*Pelodera*	1	7	15	2	19
*Rhabditis*	1	69	64	49	43
*Protorhabditis*	1	15	120	63	178
Panagrolaimidae	—	—	—	—	—
*Panagrolaimus*	1	3	3	11	36
Mononchida	—	—	—	—	—
Monhysteridae	—	—	—	—	—
*Prismatolainus*	1	47	183	55	166
*Geomonhystera*	2	5	12	6	16
**Fungivores**	—	—	—	—	—
Aphelenchida	—	—	—	—	—
Aphelenchidae	—	—	—	—	—
*Aphelenchus*	2	37	110	32	167
Tylenchida	—	—	—	—	—
Aphelenchoididae	—	—	—	—	—
*Aphelenchoides*	2	2	0	0	5
Anguinidae	—	—	—	—	—
*Ditylenchus*	2	0	0	1	32
**Predators/Omnivores**	—	—	—	—	—
Dorylaimida	—	—	—	—	—
Dorylaimidae	—	—	—	—	—
*Aporcelaimus*	5	9	17	1	16
*Eudorylaimus*	4	24	64	33	73

^a^Corresponding to their positions along the colonizer-persister continuum of their life-histories. ^b^Combination of *Streptomyces rubrogriseus* HDZ-9-47 and cabbage biofumigation. ^c^HDZ-9-47 alone. ^d^Soil biofumigation with cabbage. ^e^Equal volume of water without nematicide or biocontrol agent.

**Figure 3:**
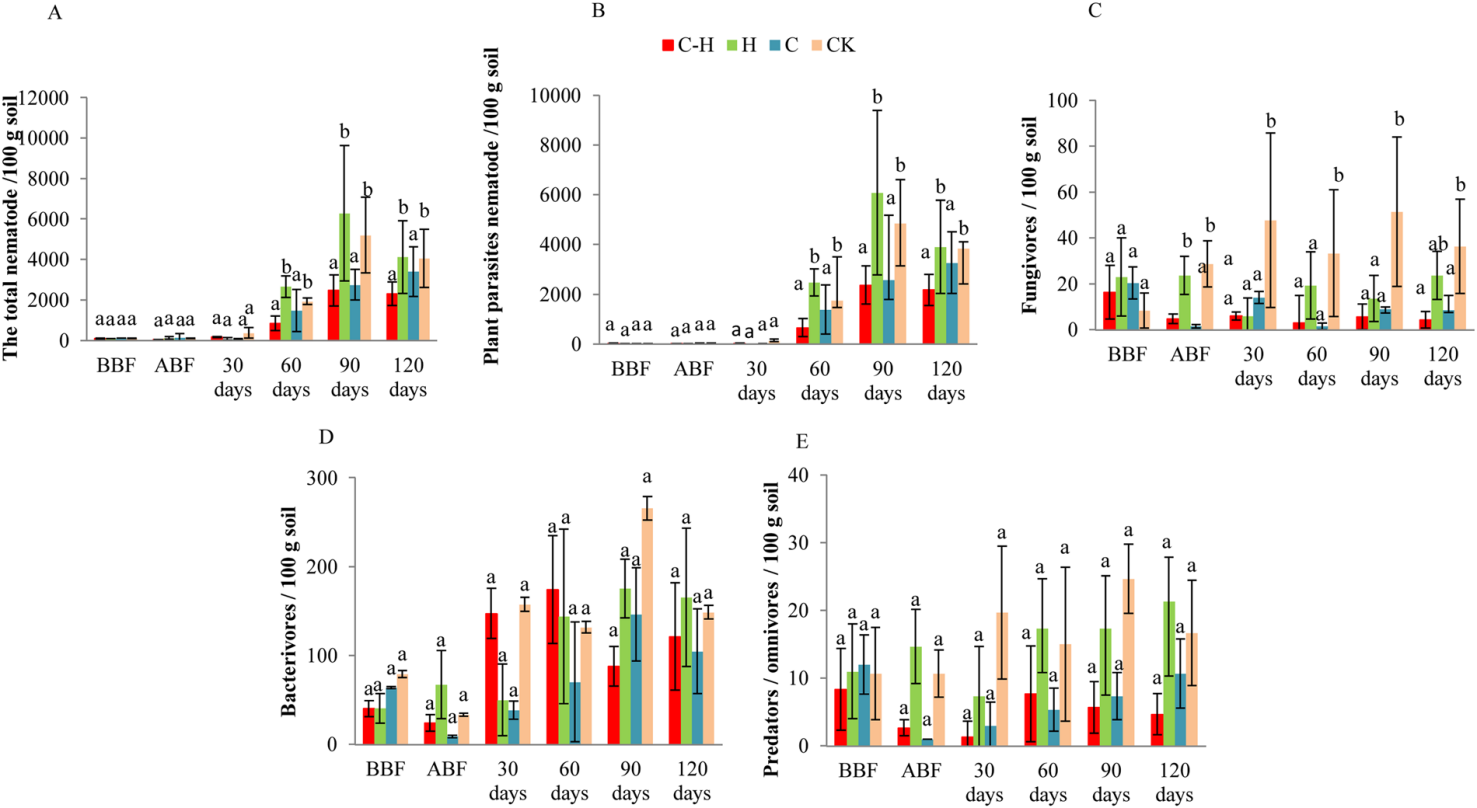
Effect of the combination of HDZ-9-47 and biofumigation on the abundance of soil total nematodes (**A**), plant parasites (**B**), fungivores (**C**), bacterivores (**D**), and predators/omnivores (**E**) at different sampling dates. C-H: combination of *Streptomyces rubrogriseus* HDZ-9-47 and biofumigation with cabbage. H: application of HDZ-9-47 alone. C: soil biofumigation with cabbage. BBF and ABF: before- and after biofumigation, respectively. Error bars represent standard deviation. The different letters on each bar within same sampling time represent significant differences at the 0.05 level based on Tukey’s multiple comparison test.

The abundance of total nematodes, plant parasites and bacterivores did not significantly differ among treatments before and immediately after biofumigation (*P* > 0.05, Fig. 3). However, the abundance of fungivores was significantly reduced after biofumigation (*P* < 0.05, Fig. 3c). The abundances of plant parasites in the combination treatment and biofumigation alone treatment were significantly lower than those in HDZ-9-47 alone treatment and the untreated control at 30 d, 60 d, 90 d, and 120 d after transplanting, respectively (*P* < 0.05, Fig. 3B). And the abundances of fungivores in application HDZ-9-47 and biofumigation alone and combination treatments were significantly lower than those in the untreated control at 30 d, 60 d, 90 d, and 120 d after transplanting (*P* < 0.05, Fig. 3C). The abundances of bacterivores and predators/omnivores were not significantly affected by any treatment (*P* > 0.05, Fig. 3D,E).

**Figure 4:**
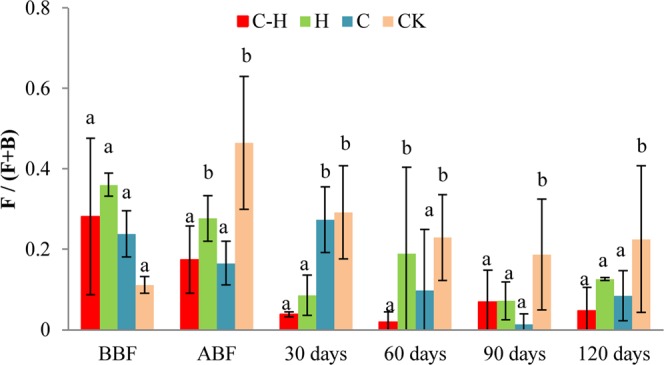
Effect of the combination of HDZ-9-47 and biofumigation on the ratio of fungivore to fungivore plus bacterivore, *F/(F* + *B)*. C-H: combination of *Streptomyces rubrogriseus* HDZ-9-47 and biofumigation with cabbage. H: application of HDZ-9-47 alone. C: soil biofumigation with cabbage. BBF and ABF: before- and after biofumigation, respectively. Error bars represent standard deviation. The different letters on each bar within same sampling time represent significant differences at the 0.05 level based on Tukey’s multiple comparison test.

The ratio of fungivores to fungivores plus bacterivores [*F/(F* + *B)*] indirectly reflects organic matter decomposition and carbon and nitrogen mineralization in the soil^19^. The combination treatment significantly reduced *F/(F* + *B)* relative to the untreated control immediately after biofumigation(ABF) and at 30 d, 60 d, 90 d, and 120 d after transplanting (*P* < 0.05, Fig. 4). The results suggest that the combination treatment accelerates organic matter decomposition and nutrient turnover in the soil.

**Figure 5:**
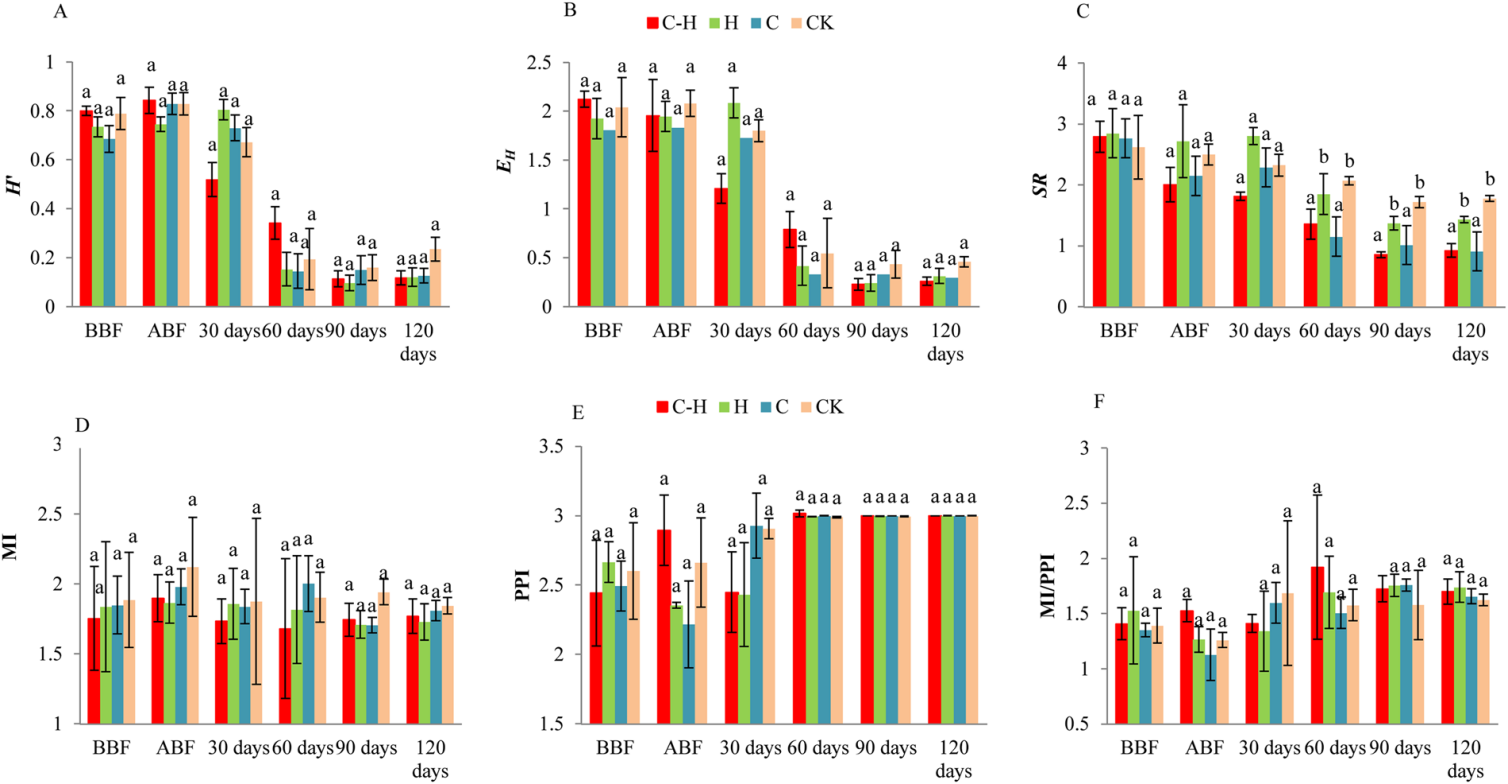
Effect of combination treatment of HDZ-9-47 and biofumigation on the Shannon diversity (*H*′) (**A**), Pielou evenness (*E*_*H*_) (**B**), Margalef richness (*SR*) (**C**), and maturity indices of free-living nematodes (*MI*, **D**), plant parasitic nematodes (*PPI*, **E**) and *MI*/*PPI* (*PPI*, **F**) at different sampling dates. C-H: combination of *Streptomyces rubrogriseus* HDZ-9-47 and biofumigation with cabbage. H: HDZ-9-47 alone. C: soil biofumigation with cabbage. BBF and ABF: before- and after biofumigation, respectively. Error bars represent standard deviation. The different letters on each bar within same sampling time represent significant differences at the 0.05 level based on Tukey’s multiple comparison test.

The Shannon diversity (*H*′), Pielou evenness (*E*_*H*_), and Margalef richness (*SR*) indices for the soil nematodes decreased with sampling time in all treatments (Fig. 5). *H*′ for the combination treatment was significantly lower than that for the untreated control at 120 d after transplanting (*P* < 0.05, Fig. 5A). *SR* was also significantly lower for the combination treatment than the untreated control at 60 d, 90 d, and 120 d after transplanting (*P* < 0.05, Fig. 5C). However, *E*_*H*_ was not significantly affected by the combination treatment relative to the control (*P* > 0.05, Fig. 5B).

The maturity index (*MI*) represented the nematode community structure. *MI* is a measure of disturbance. *MI* decreases with increasing environmental disturbance. The plant parasitic index (*PPI*) increases with agricultural enrichment. Nutrient enrichments could reduce *PPI*/*MI*. In our experiment, however, *MI*, *PPI*, and *MI/PPI* were not significantly affected by any treatment at any sampling time (*P* > 0.05, Fig. 5D–F).

## Discussion

The combined application of *S. rubrogriseus* HDZ-9-47 and biofumigation had superior efficacy against *M. incognita* compared with either treatment alone^9^. In our early report, the reduction rates of root-knot index in combined application of HDZ-9-47 with biofumigation, HDZ-9-47 alone, and biofumigation alone treatments were 87.1, 45.7, and 61.4 at 90 d post transplantation, respectively. And the reduction percentages of J2s density in those treatments were 91.0, 69.7, and 77.8, respectively^9^. In the present study, the combined application reduced root galls by 41% and its control efficacy was greater than each treatment alone. This finding corroborates those of previous reports.

The soil microbial community plays an important role in disease control. Beneficial soil microbes may help suppress plant pathogens^20,21^. Wang *et al*. reported that integrating biofumigation with antagonistic microorganisms controls *Phytophthora* blight by regulating soil bacterial community structure^15^. In this study, we hypothesized that the gains in efficacy against *M. incognita* realized by the combination of *S. rubrogriseus* HDZ-9-47 and biofumigation are associated with their effects on the soil microbial community. Cultivation-based analyses showed that at 30 d and 60 d after transplanting, the soil culturable bacterial and actinomycetes densities increased in response to HDZ-9-47, biofumigation, and especially the combination of the two. The culturable actinomycetes and bacterial densities were negatively correlated with root gall score. Our PCR-DGGE analysis showed the bacterial bands BHA4 (*Streptomyces* sp.) only appeared in the combination treatment. Actinomycetes may produce various secondary metabolites with nematicidal activity to help control plant parasitic nematodes. *Streptomyces* is the major actinomycetes genus. Its member species control plant parasitic nematodes by antagonism or parasitism^3,4^. Sun *et al*. (2006) reported that a total of 52 actinomycetes isolates were obtained from eggs and females of *Meloidogyne* spp. Most of these isolates could parasitize eggs of *Meloidogyne hapla*, inhibit egg hatch, and kill second-stage juveniles (J2s) *in vitro*. In the combination treatment, the fungal band FHA6 (*Rhizoctonia* sp.) disappeared. Earlier studies showed that most *Rhizoctonia* sp. are globally distributed soil-borne fungi which can infect many economically important field crops^22^. Cultivation-based analysis showed that the fungal density was positively correlated with the root gall number and decreased in the HDZ-9-47, biofumigation, and combination treatments relative to the control. These results suggest that the combination of *S. rubrogriseus* HDZ-9-47 and biofumigation promoted the reproduction of some fungal species. The combination treatment enriched beneficial microbes and reduced certain soil-borne fungal phytopathogens, thereby enhancing the efficacy of both *S. rubrogriseus* HDZ-9-47 and biofumigation against *M. incognita*. A previous report indicated that the abundance of beneficial microbes was higher in response to the combined application of lime, ammonium bicarbonate, and bioorganic fertilizer than the control treatment^23^. Certain studies observed increases in bacterial densities and decreases in fungal densities after the introduction of biocontrol agents like *Beauveria bassiana* or *Pseudomonas fluorescens* 2P24^24,25^. Ascencion *et al*. found that the soil fungal density was positively correlated with the incidence of *Rhizoctonia solani* damping-off disease. In contrast, the soil actinomycetes density was negatively correlated with damping-off after biofumigation with *Brassica*^26^.

The soil nematode community is an indicator of environmental changes caused by agricultural practices^27,28^. In this study, the abundance of fungivores was significantly reduced after biofumigation (*P* < 0.05). Wang G, *et al*. (2009) also found that Brassica biofumigants reduced the fungivores in the soil. Relative to the control in the present study, the abundance of plant parasites was not significantly reduced immediately after biofumigation (*P* > 0.05), but was significantly decreased in response to the combination and biofumigation alone treatments at 30 d, 60 d, 90 d, and 120 d after transplanting. However, the abundances of omnivorous/predaceous nematodes were not affected by this treatment. Therefore, the decline in plant parasites was not attributed to toxic volatile compounds produced by biofumigation or top-down control by predator nematodes^29^. Gruver *et al*. found that biofumigation did not influence the abundances of omnivores/predators^30^. The ratio of fungivores to fungivores plus bacterivores [*F/(F* + *B)*] indirectly reflects organic matter decomposition and carbon and nitrogen mineralization in the soil^19^. The combination treatment significantly reduced *F/(F* + *B)* compared with the untreated control. Therefore, there may have been high organic matter decomposition rates and fast nutrient turnover in this treatment. The nematode diversity index has been commonly used to assess the impact of human intervention on the nematode community^10^. Our previous trials in Tong zhou district of Beijing showed that the combined application of HDZ-9-47 with biofumigation reduced the abundances of total nematodes and plant parasites, and decreased the *SR* of the soil nematodes (*P* < 0.05). And the combination treatment had no significant effect on *MI*, *PPI*, or *MI/PPI* (*P* > 0.05). Our current field trial presented with similar results. Earlier studies reported that the nematode diversity index increased in fields treated with biofumigation relative to the control^31,32^. However, this effect was not observed in the present study. In contrast, *H’*, *SR*, and *E*_*H*_’ decreased relative to the control in the combination treatment at 120 d after transplanting, which is the late stage of tomato growth. This delayed decrease in biodiversity may be explained by the fact that biofumigation alters the soil microbe communities used as food by nematodes. This effect may influence the nematode density.

Biocontrol agents or biofumigation may have short- or long-term effects on soil microbial communities^10^. Some researchers reported only transient effects on soil microbial communities following inoculation with biocontrol agents like *Pseudomonas fluorescens* 2P24, *P. fluorescens* CPF10, and *Bacillus subtilis* Jdm2^27,33,34^. The combination of HDZ-9-47 and biofumigation only had an impact on the soil microbial community at the early stages of tomato growth. Our results also showed that the soil microbe community was mainly influenced by plant growth.

Biocontrol agent colonization in the soil is essential for efficacy^35^. The PCR-DGGE analysis identified a strong band (*Streptomyces*) corresponding to the HDZ-9-47 isolate which was visible in all HDZ-9-47 treatments at 30 d and 120 d after transplanting. Therefore, we inferred that HDZ-9-47 may colonize in the soil.

In addition, we applied PCR-DGGE approach to investigate the soil microbial community in this work. PCR-DGGE has been the most applied technique for microbial community studies for a long time; its advantages include the relatively low cost of instrumentation, the possibility of processing several samples altogether and the relative ease of use^36^. However, PCR-DGGE suffers from a number of drawbacks, the main ones being represented by low resolution power, background noises and difficulties in extrapolating quantitative data by the analysis of band intensities^36^. In this work, nested PCR was used to determine the fingerprints of fungi community. The overamplification of 60 cycles may generate the prevalence of some bands over other bands that do not amplify so easily. PCR-DGGE was only used as a tool for analyzing comparative community structure, not as a means of quantifying α-diversity^37^. To more deep understand the effect of combined application of HDZ-9-47 with soil biofumigation on soil microbial diversity, high-throughput sequencing technologies (HTS) should be employed in future investigation.

In conclusion, combined application of *S. rubrogriseus* HDZ-9-47 with biofumigation had significant effects on the soil microbial and nematode communities at the early stages of tomato growth, which contribute to control *M. incognita* through direct and indirect effects. This study provides new insights into the reason of improvement efficacy of the combination against *M. incognita*. In addition, the combination of *S. rubrogriseus* HDZ-9-47 and biofumigation only have short-term effects on soil microbial communities. To maximize the potential of *S. rubrogriseus* HDZ-9-47 and biofumigation, future work is required to elucidate the effects of biofumigation on *S. rubrogriseus* HDZ-9-47 colonization.

## Materials and Methods

### HDZ-9-47 liquid culture

*Streptomyces rubrogriseus* HDZ-9-47 was obtained from the Institute of Microbiology of the Chinese Academy of Science and deposited at the China General Microbiological Culture Collection Center as CGMCC 2878. The isolate was cultured in a medium consisting of 1.05% corn flour, 1.825% bean flour, 0.22% MgSO_4_·7H_2_O, 0.15% K_2_HPO_4_·3H_2_O, 0.1% CaCO_3_, and 0.0238% MnSO_4_, w/v (pH 7.3). This formulation was described by Jin *et al*.^9^.

### Field conditions and experiment design

Trials were conducted in a protected field (length 90.0 m; width 5.5 m) in the Chang ping district, Beijing, China (41 °2 ′N, 116 °2′E) in springtime 2014. The field was naturally infested with *M. incognita*. The soil was a calcareous sandy loam with pH 7.13 ± 0.04. It contained 15 g kg^−1^ organic matter, 1 g kg^−1^ total nitrogen, 143.9 g kg^−1^ available potassium, and 207.5 g kg^−1^ available phosphorus. The daily air temperature ranged from 15-38 °C. The field was continuously cultivated with tomato (*Solanum lycopersicum*) and treated with fosthiazate to control root knot nematode for 2- y before the start of our trials.

Thirty-day-old tomato seedlings Cv. Zhefen 702 (susceptible to *M. incognita*) were transplanted into the field after soil treatment by *S. rubrogriseus* HDZ-9-47, biofumigation or their combination. The treatments were designed as follows: (1) HDZ-9-47 alone: a 200 ml cultures containing 10^12^ HDZ-9-47 spores was drenched into the planting hole (H); (2) biofumigation: cabbage residue and NH_4_NO_3_ (Tianjin Tongxin Chemical Co., Ltd., Tianjin, China) were incorporated into the top 20 cm of the soil at a rate of 3.5 kg m^−2^ and 0.1 kg m^−2^, respectively. Then the soi1 was irrigated to maximum field capacity with a drip irrigation system and covered with transparent polythene film (0.2 mm thickness) for 20 d (C), (3) HDZ-9-47 combination with soil biofumigation: a 200 ml cultures containing 10^12^ HDZ-9-47 spores was drenched into the planting hole after the soil was biofumigated with 3.5 kg/m^2^ cabbage (C-H), (4) untreated control (CK). Details please see the methods descripted by Jin *et al*.^9^.

The treatments were arranged in a randomized complete block design (RCBD) with three replicates per treatment. Each replicate (length 5.5 m; width 1.5 m) consisted of ≥32 plants. The protected field was irrigated by a linear drip irrigation system as required and fertilized in accordance with local growing practices.

### Data collection

The soil was sampled 0–20 cm below the rhizosphere surface using a soil corer (diameter: 2 cm; Soil Sampler Inc., Johns Creek, GA, USA) before and after biofumigation at 30 d, 60 d, 90 d, and 120 d after transplanting. The samples were stored at −80 °C or at 4 °C for the soil microbial and the soil nematode community tests, respectively. At 90 d after transplanting, five plants and their rhizosphere soils were collected per treatment. Root galls on nematode-infected plants were assessed with a 0–10 rating scale according to Bridge and Page^38^. Nematodes in 100 cm^3^ tomato rhizosphere soil, including *M. incognita* juveniles, were extracted by a modified salt-centrifugal-flotation technique^39^. The recovered nematodes were observed and counted under a compound- or stereoscopic microscope (SZ61; Olympus Corp., Tokyo, Japan).

### Cultivation-based analyses of microbial densities

Culturable microbial densities were determined by cultivation-based analyses^40^. Ten grams of rhizospheric soil was mixed with 90 mL of sterile water in a 200-mL flask by shaking on a rotary shaker for 30 min at 180 rpm. Serial 10× dilutions were then prepared down to 10^−7^. Then 0.1-mL aliquots of the appropriate dilutions were spread on the corresponding media in triplicate. Fungi were cultured on Potato Dextrose Agar (PDA) supplemented with 25 mg L^−1^ streptomycin sulfate (Sigma Aldrich Chemical, Germany) at 28 °C for 7 d. Actinomycetes were cultured on Gauze’s Agar supplemented with 50 mg L^−1^ potassium dichromate (Sigma Aldrich Chemical, Germany) at 28 °C for 7 d. The bacteria were cultured on Luria Bertani Agar (LB) at 37 °C for 48 h. The colony-forming units (CFU) of the fungi, actinomycetes, and bacteria were then counted.

### DNA extraction from soil

Total soil DNA was extracted with a PowerMax^®^ Soil DNA Isolation Kit (MoBio Laboratories, Inc., Solana Beach, CA, USA) following the manufacturer’s protocol. The DNA was stored at −20 °C until later use.

### Polymerase chain reaction (PCR) and denaturing gradient gel electrophoresis (DGGE)

The soil bacterial and fungal community structures were determined by PCR-DGGE. The bacterial 16 S rDNA fragment was amplified with the 338f-GC clamp (5′-CCTACGGAGGCAGCAGCGCCCGGGGCGCGCCCCGGGGCGGGGCGGGGGCGCGGGGGG-3′)/518r (5′-CCTACGGGAGGCAGCA G-3′) primer pair^41^. PCR was run in a 50-μL volume consisting of 0.5 μL *rTaq* DNA polymerase (TaKaRa Bio Inc., Kusatsu, Shiga, Japan), 5 μL of 10× PCR buffer (TaKaRa Bio Inc., Kusatsu, Shiga, Japan), 4 μL dNTP mix (TaKaRa Bio Inc., Kusatsu, Shiga, Japan), 5 ng extracted soil DNA, 20 μM of each primer, and 38.5 μL ddH_2_O. The thermal cycling program was as follows: initial denaturation at 94 °C for 5 min, then 30 cycles of 30-s denaturation at 94 °C, then 30 s annealing at 55 °C, and 30 s extension at 72 °C. The final extension was 10 min at 72 °C. The mixture was cooled to 4 °C.

The fungal internal transcribed spacer (ITS) fragment was amplified before DGGE analysis using a nested PCR approach with the primer pairs ITS1f (5′-CTTGGTCATTTAGAGGAAGTAA-3′)/ITS4 (5′-TCCTCCGCTTATTGATATGC-3′) and ITS1f-GC clamp (5′-CGCCCGCCGCGCGCGGCGGGCGGGGCGGGGGCACGGGGGGCTTGGTCATTTAGAGGAAGTAA-3′)/ITS2 (5′-GCTGCGTTCTTCATCGATGC-3′)^42^. First-round PCR was performed in a 25-μL volume consisting of 0.25 μL *ExTaq* DNA polymerase (TaKaRa Bio Inc., Kusatsu, Shiga, Japan), 2.5 μL of 10× *Ex Taq* Buffer (TaKaRa Bio Inc., Kusatsu, Shiga, Japan), 2 μL dNTP mix (TaKaRa Bio Inc., Kusatsu, Shiga, Japan), 5 ng extracted soil DNA, 20 μM ITS1f, 20 μM ITS4, and 18.25 μL ddH_2_O. The thermal cycling program was as follows: initial denaturation at 94 °C for 5 min, then 35 cycles of 1 min denaturation at 94 °C, then 45 s annealing at 50 °C, and 1 min extension at 72 °C. The final extension was 10 min at 72 °C. The products served as templates for the second PCR. The reaction mixture for the second PCR was the same as that for the first except ITS1f-GC/ITS2 was used instead of ITS1f/ITS4. The PCR conditions were the same as those described for the first PCR except 25 cycles were run instead of 35.

DGGE was conducted with a DCode^TM^ Universal Mutation Detection System (Bio-Rad Laboratories Inc., Hercules, CA, USA). Twenty microliters of PCR products containing 200 ng DNA were loaded onto 8% acrylamide gel with a linear chemical gradient ranging from 35–55% denaturant, where 100% denaturant = 7 M urea + 40% formamide)^43^. The polyacrylamide gels were prepared with a Model 475 Gradient Delivery System (Bio-Rad Laboratories Inc., Hercules, CA, USA). The gel electrophoresis was run in 1× TAE buffer (40 mM Tris–acetate and 1 mM EDTA; pH 8.0) for 4 h at 60 °C and 150 V for bacteria and for 17 h at 60 °C and 100 V for fungi. The gels were stained with silver according to the protocol of Radojkovic and Kušic^44^ and captured with a Fluor-S Multi-imager (Bio-Rad Laboratories Inc., Hercules, CA, USA).

### Sequence analyses

The intense DGGE bands found in all treatments or bands only found in the combination treatment or CK treatment were excised from the gel with a sterile scalpel under UV illumination. The DNA was eluted overnight at 4 °C in 20 μL sterile water^45^. The excised DNA was then re-amplified with 338 f /518r and ITS1f/ITS2 as described above. After purification, the DNA fragments were ligated to the pMD18-T cloning vector (TaKaRa Bio Inc., Kusatsu, Shiga, Japan) and transformed into *Escherichia coli DH5α* (GenStar Biosolutions Co. Ltd., Beijing, China) according to the manufacturer’s instructions. Three positive clones were randomly selected per band for DNA sequencing in a Qingke Biotech (Qingke Co. Ltd., Beijing, China). The resulting sequences were compared by BLAST search (http://www.ncbi.nlm.nih.gov/blast/Blast.cgi) with those in public databases.

### Isolation and identification of nematode

Nematodes were extracted from 100 cm^3^ of rhizospheric soil by the modified salt-centrifugal-flotation technique^40^. The extracted nematodes were immediately fixed according to the method described by Seinhorst^46^. The fixed nematodes were observed under a stereoscopic microscope (SZ61; Olympus Corp., Tokyo, Japan) and identified to the genus level with the identification keys of Yin^47^. Nematodes were assigned to trophic groups (plant parasites, fungivores, bacterivores, or predators/omnivores) according to the method of Yeates^48^. They were also assigned colonizer-persister (c-p) values of 1–5 corresponding to the positions of their life histories along the colonizer-persister continuum^38^.

### Statistical analyses

Data were analyzed in SPSS v. 15.0 (IBM Corp., Armonk, NY, USA). One-way ANOVA followed by Tukey’s post hoc test was run to identify significant differences between treatments. Pearson’s correlation coefficients were determined for bivariate correlations. Permutational multivariate analysis of variance (PERMANOVA; PRIMER-E/Quest Research Ltd., Auckland, NZ) was used to evaluate statistical significance between DGGE profiles. All statistical tests were performed using 0.05 as the significance level. Soil microbial and nematode population data were log-transformed then subjected to ANOVA.

Banding patterns of the DGGE profile were analyzed in Quantity One v. 4.6.2 (Bio-Rad Laboratories Inc., Hercules, CA, USA).

The total abundance of nematodes per trophic group and the percentage of each trophic group in the nematode community were calculated. The ratio of fungivore to fungivore plus bacterivore [*F*/(*F* + *B*)] was calculated to characterize decomposition and mineralization pathways^49^.

The nematode community structure was determined by the maturity index which was measured based on the life history strategy characteristics of the nematode taxa. The maturity indices were calculated separately for plant parasitic (*PPI*) and free-living (*MI*) families^38^ according to formula (1).

1 $${\boldsymbol{MI}}({\boldsymbol{PPI}})={\sum }_{{\boldsymbol{i}}=1}^{{\boldsymbol{n}}}{\boldsymbol{CPi}}\times {\boldsymbol{Pi}}$$

where *CPi* is the colonizer-persister (c-p) value assigned to family *i*, *Pi* is the proportion of family *i* per sample., and *n* is the total number of individuals per sample^38^.

Nematode community diversity was estimated with the Shannon diversity (*H*′), Margalef richness (*SR*), and Pielou evenness (*E*_*H*_) indices according to formulae (2), (3), and (4).

2 $${\boldsymbol{H}}^{\prime} =\displaystyle {\sum }_{{\boldsymbol{i}}={\bf{1}}}^{{\boldsymbol{s}}}{\boldsymbol{Pi}}({\bf{In}}{\boldsymbol{Pi}})$$

3 $${{\boldsymbol{E}}}_{{\boldsymbol{H}}}=\displaystyle \frac{{\boldsymbol{H}}^{\prime} }{{\bf{In}}{\boldsymbol{S}}}$$

4 $${\boldsymbol{SR}}=\frac{{\boldsymbol{S}}-{\bf{1}}}{{\bf{l}}{\bf{n}}{\boldsymbol{S}}}$$

where *Pi* is the proportion of family *i* in the total nematode community and *S* is the number of individuals in family *i*^50^.

## Supplementary information

Supplementary information
