# Supplementary material for: The effect of combined application of Streptomyces rubrogriseus HDZ-9-47 with soil biofumigation on soil microbial and nematode communities
Source: Sci Rep. 2019 Nov 15;9:16886. doi: 10.1038/s41598-019-52941-9 (PMC6858445; doi:10.1038/s41598-019-52941-9)
Supplement: Supplementary file 1 — Supplementary information [file 41598_2019_52941_MOESM1_ESM.pdf]

The effect of combined application of Streptomyces rubrogriseus HDZ-9-47 with soil biofumigation on soil microbial and nematode communities

Na Jin[1,2,3], Xiuliang Lu[1], Xueyan Wang[1], Qian Liu[1], Deliang Peng[2], Heng Jian[1]*

[1]Key Laboratory of Pest Monitoring and Green Management, Ministry of Agriculture, Department of Plant Pathology, China Agricultural University, Beijing 100193, China

[2]State Key Laboratory for Biology of Plant Diseases and Insect Pests, Institute of Plant Protection, Chinese Academy of Agricultural Sciences, Beijing 100193, China

[3]Department of Horticulture, Beijing Vocational College of Agriculture, Beijing 102442, China

***Corresponding author**

Correspondence and requests for materials should be addressed to H. J**. (E-mail address:** hengjian@cau.edu.cn**)**

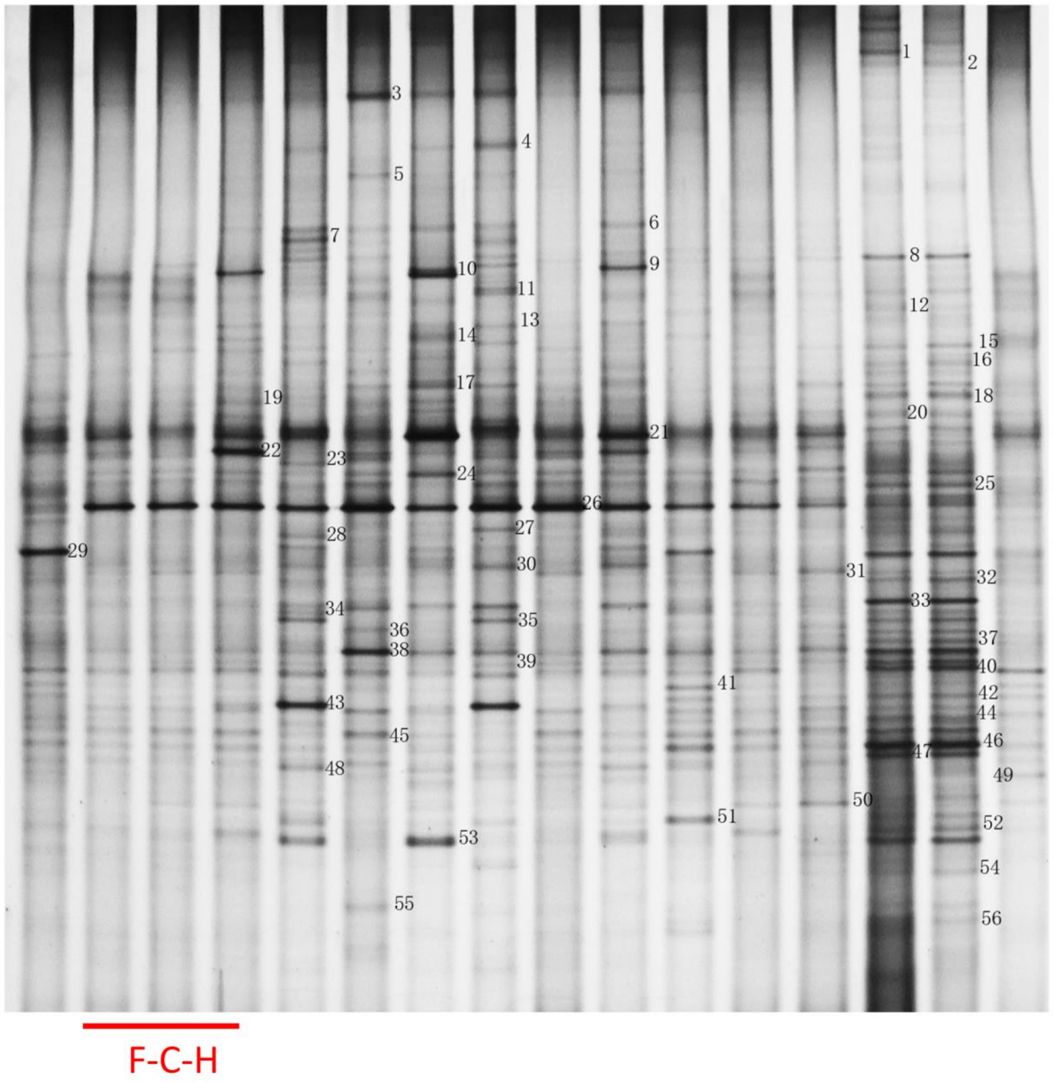

Figure 2 DGGE patterns of bacterial 16S rDNA genes (A)

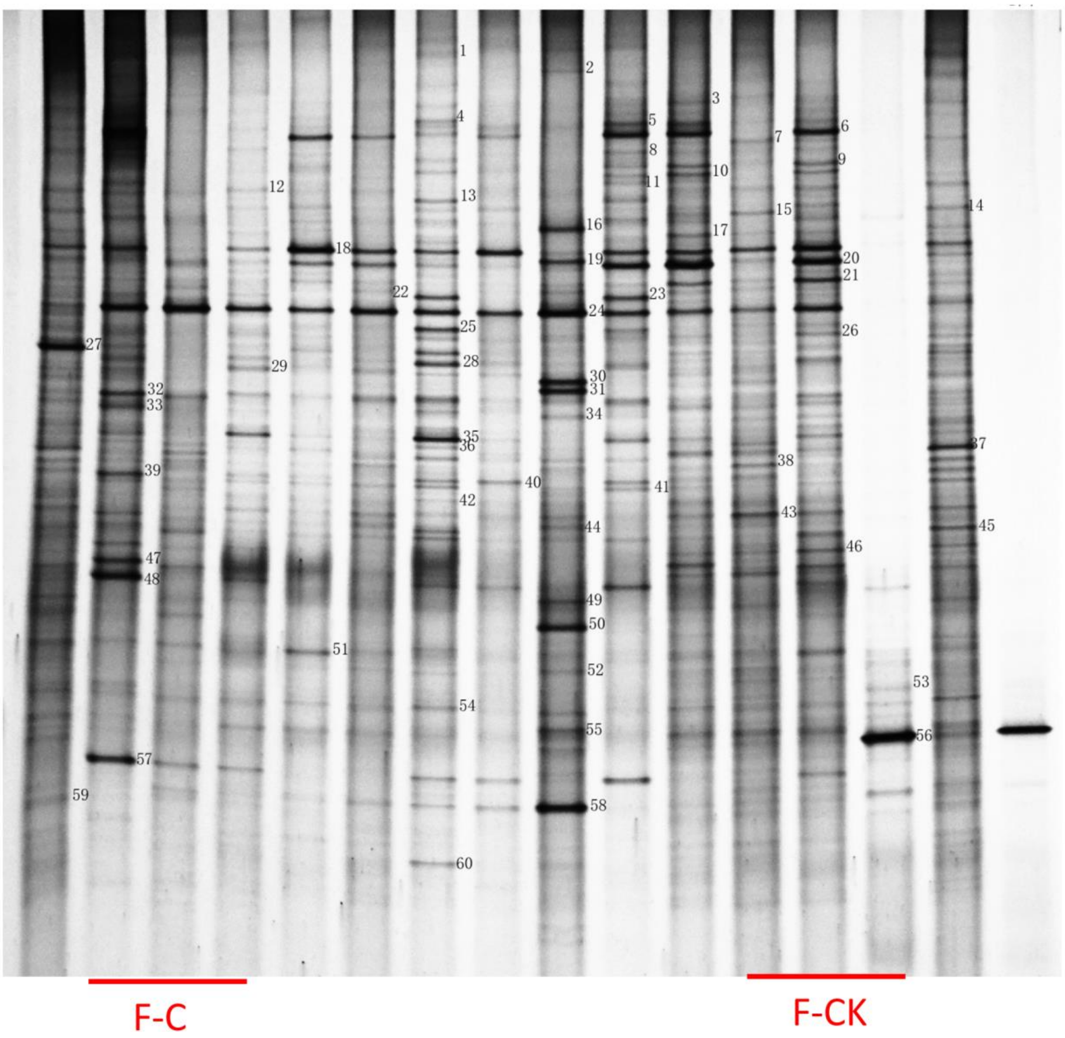

Figure 2 DGGE patterns of bacterial 16S rDNA genes (A)

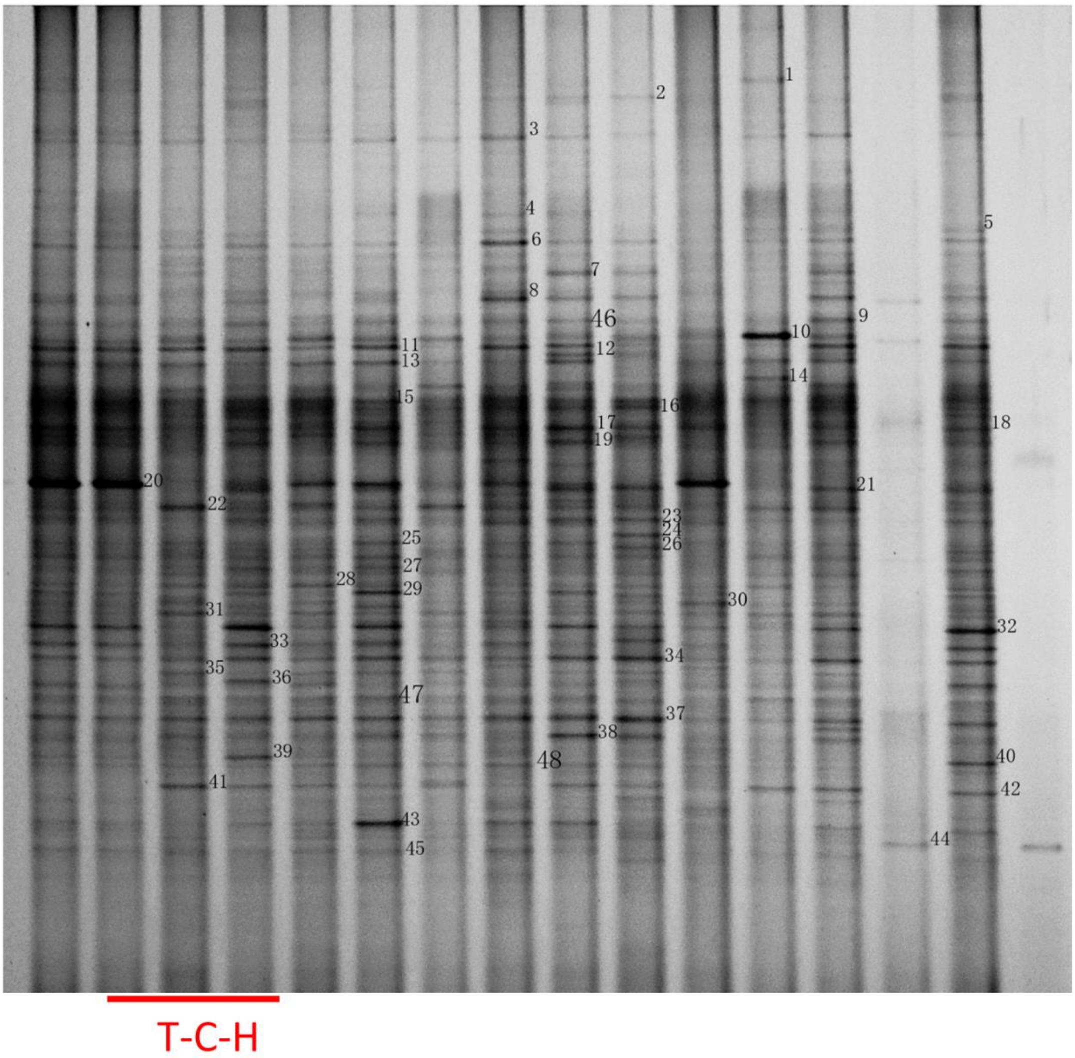

Figure 2 DGGE patterns of bacterial 16S rDNA genes (B)

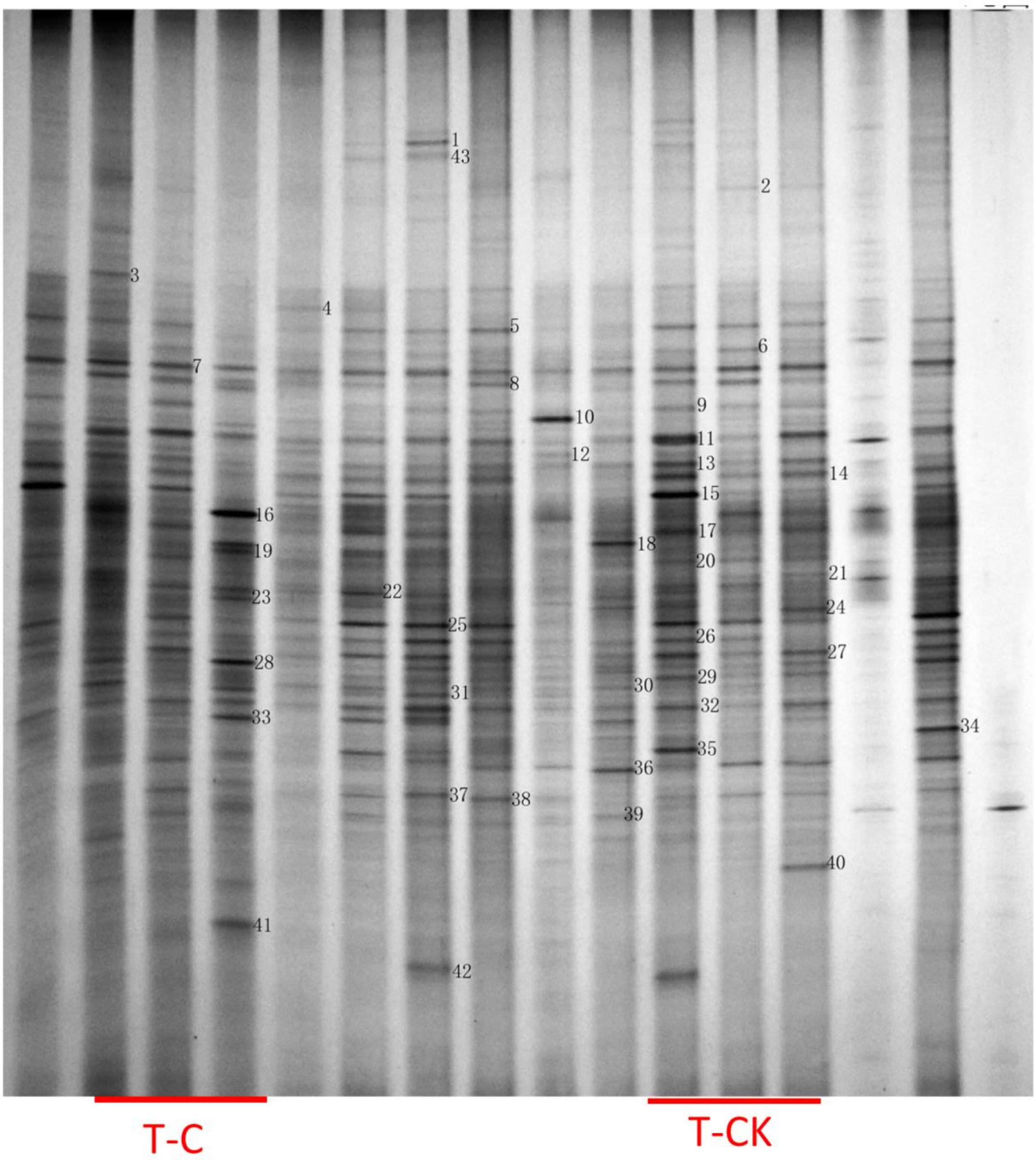

Figure 2 DGGE patterns of bacterial 16S rDNA genes (B)

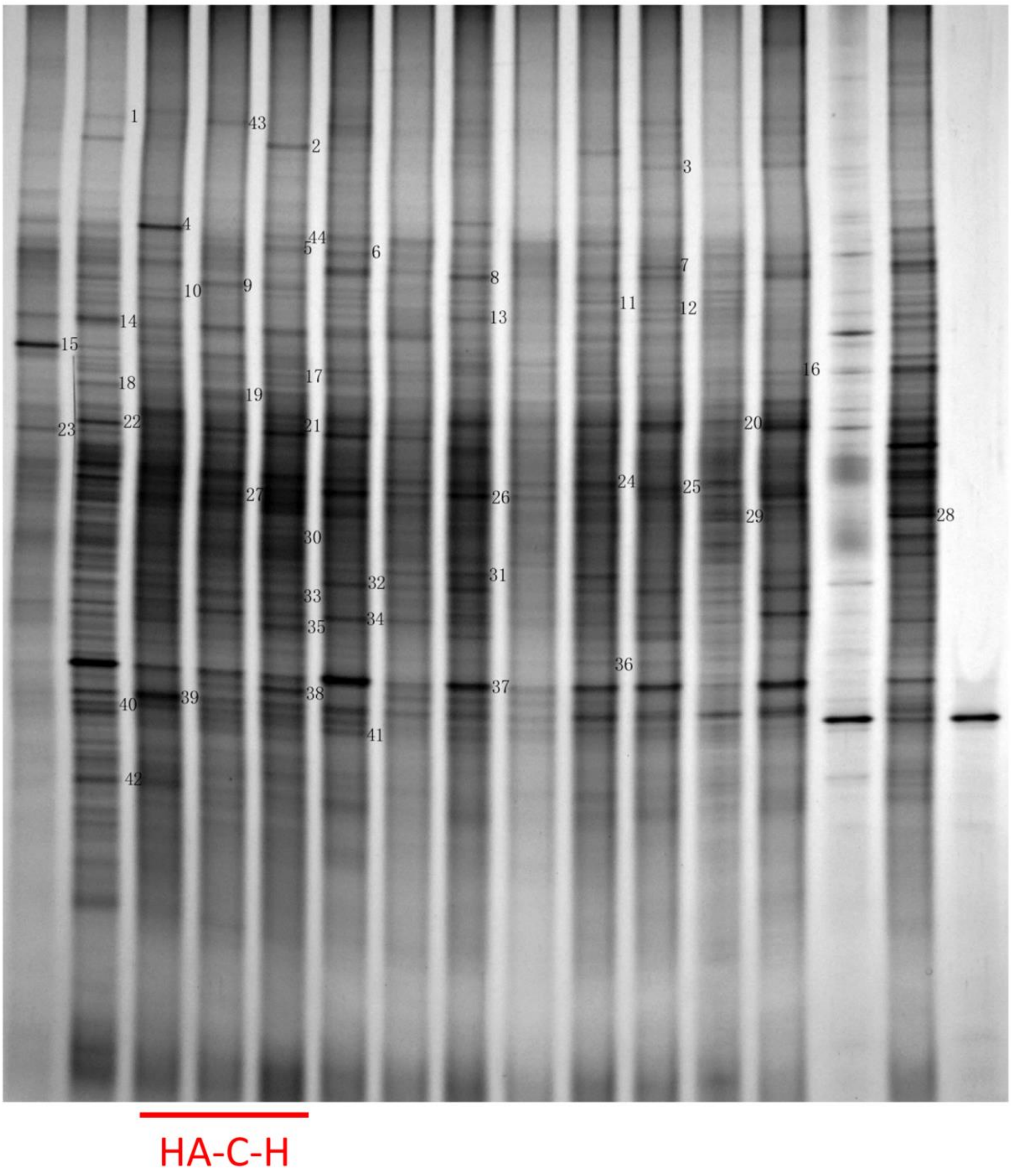

Figure 2 DGGE patterns of bacterial 16S rDNA genes (C)

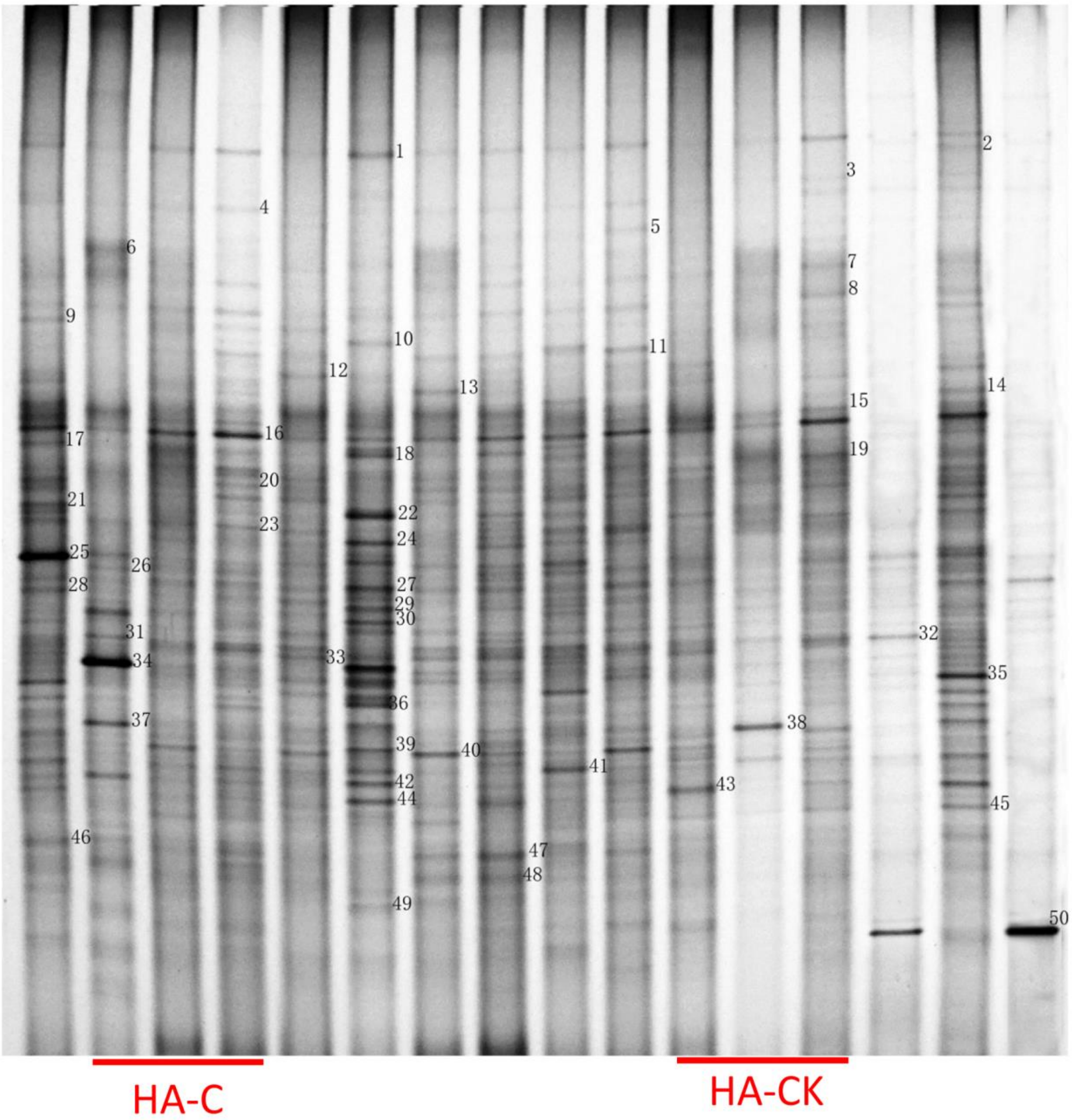

Figure 2 DGGE patterns of bacterial 16S rDNA genes (C)

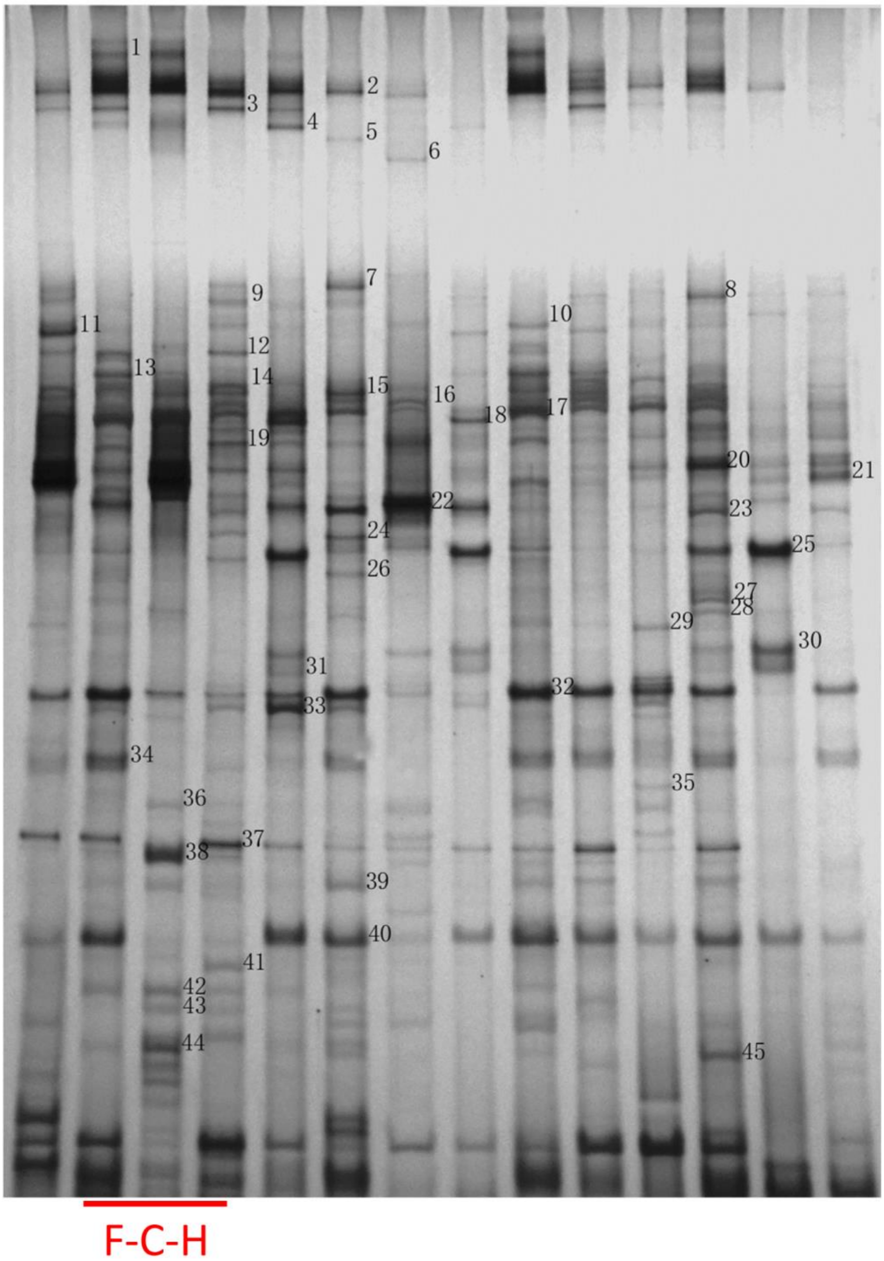

Figure 2 DGGE patterns of fungal ITS (D)

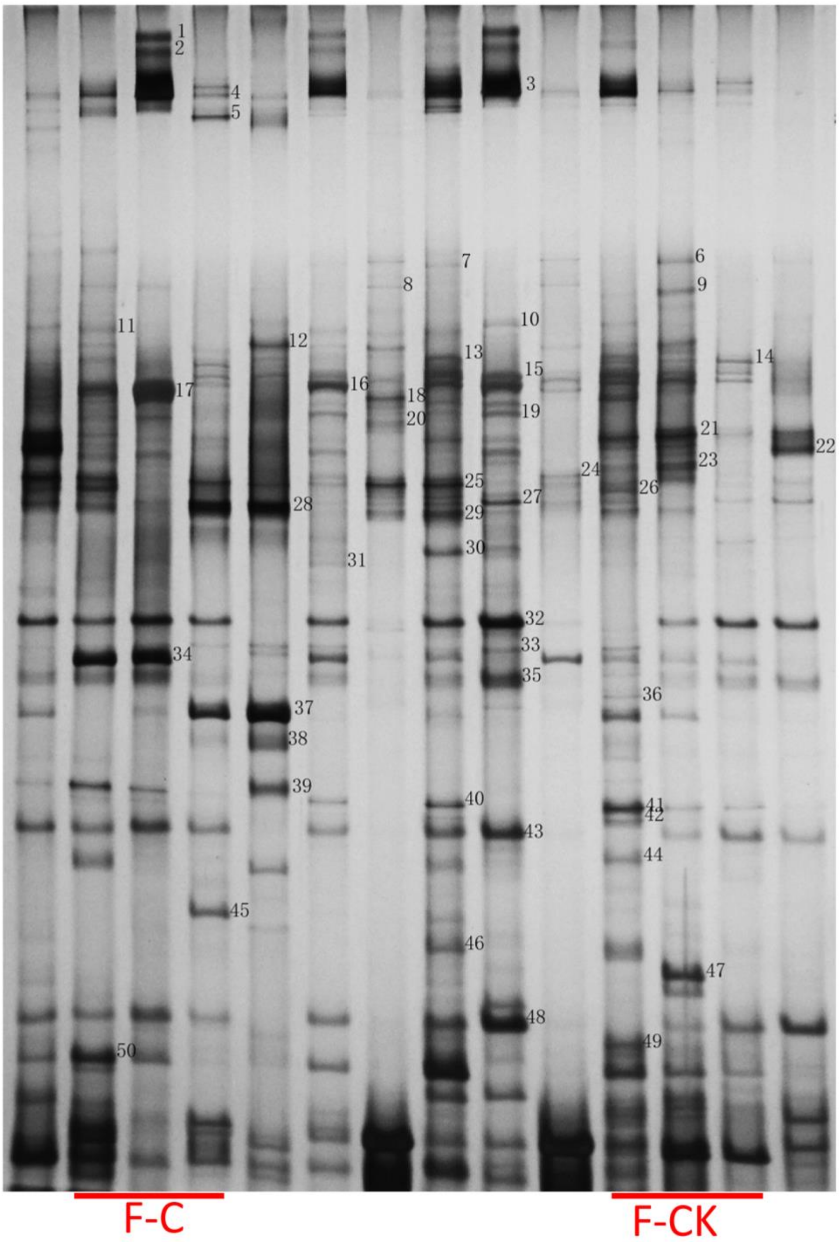

Figure 2 DGGE patterns of fungal ITS (D)

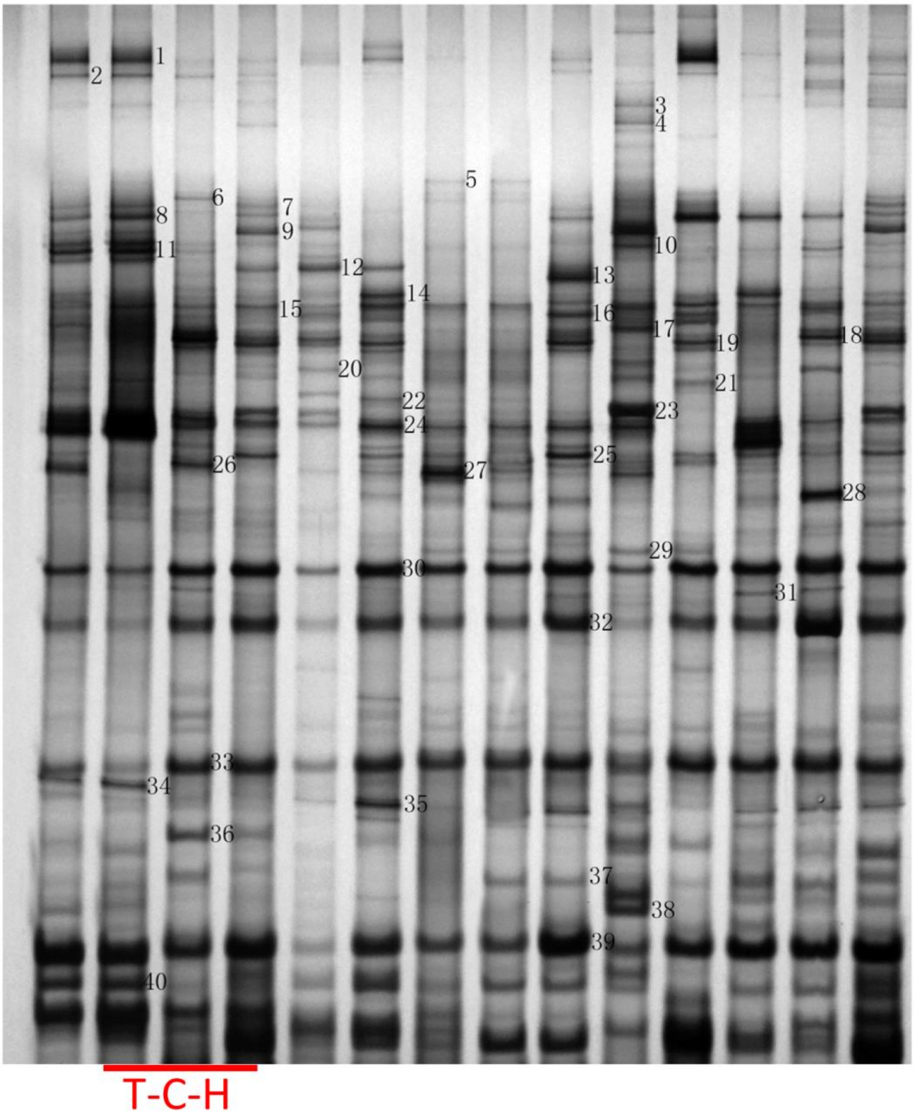

Figure 2 DGGE patterns of fungal ITS (E)

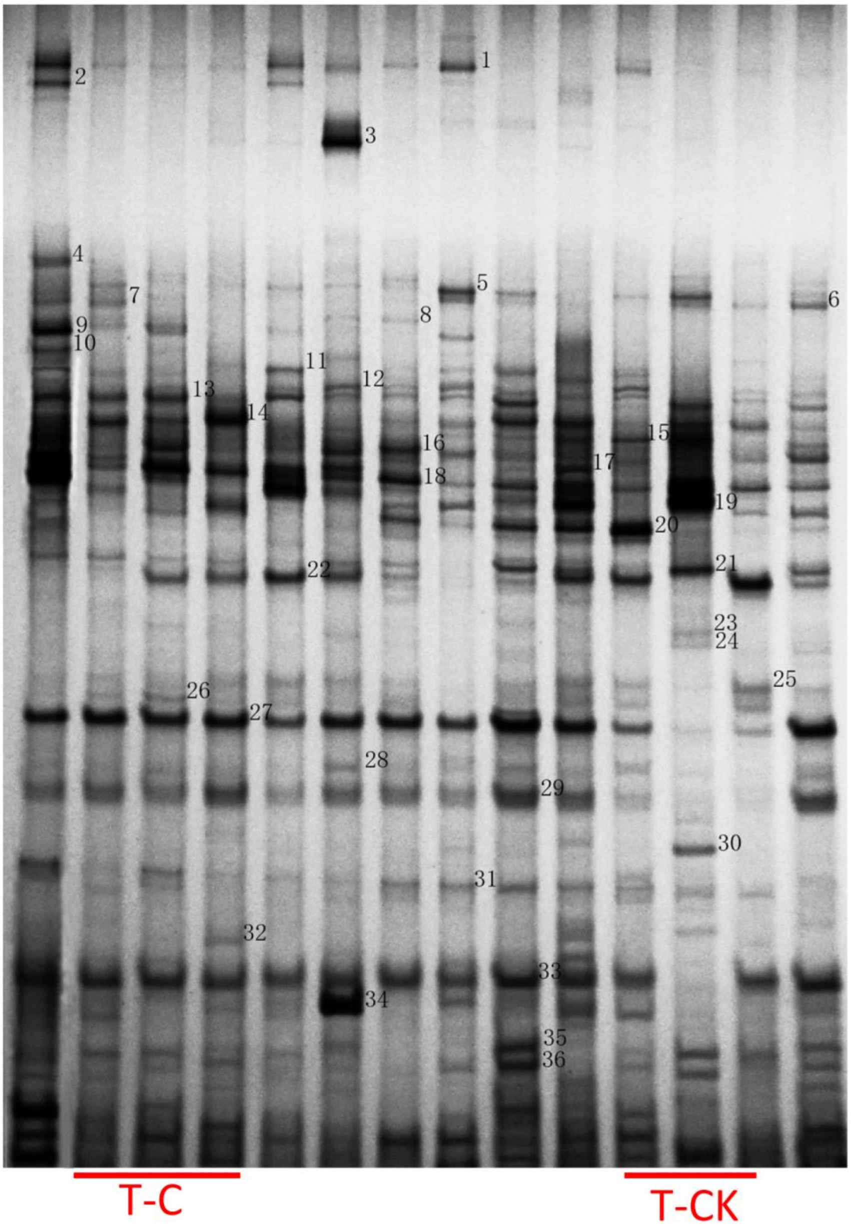

Figure 2 DGGE patterns of fungal ITS (E)

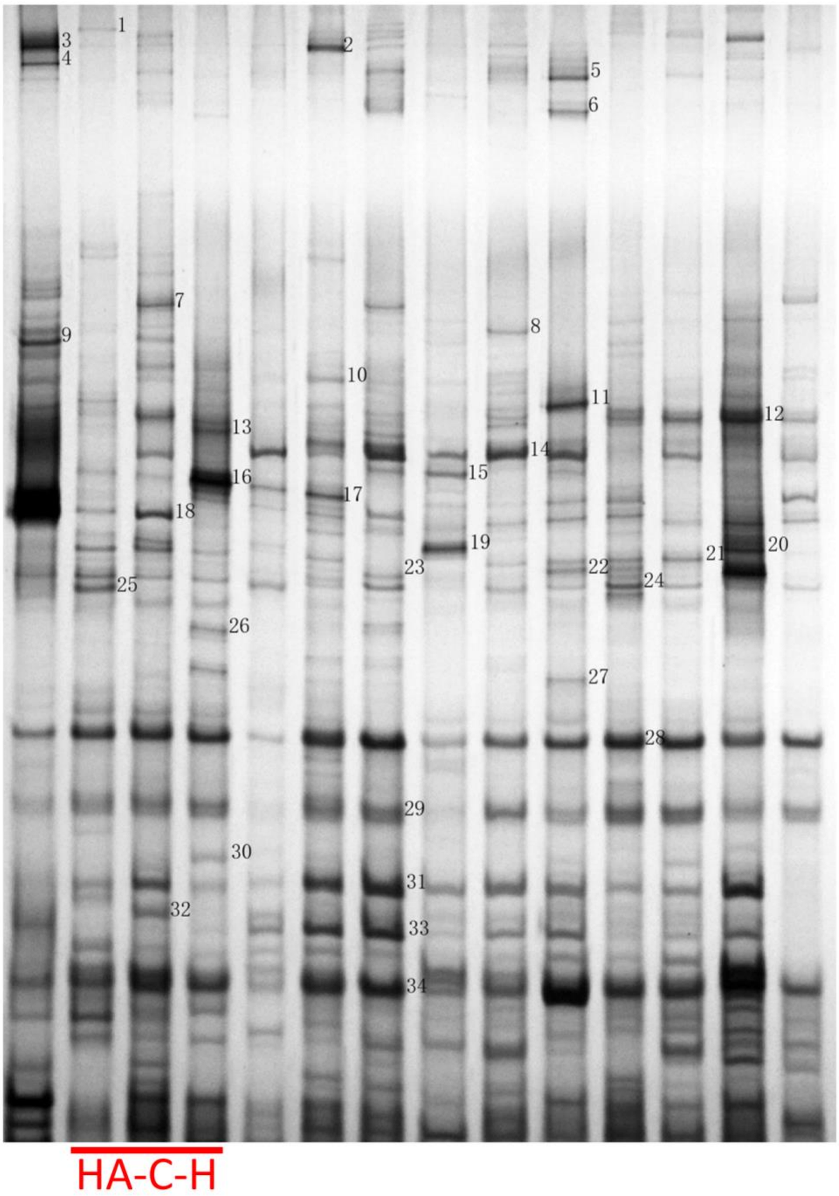

Figure 2 DGGE patterns of fungal ITS (F)

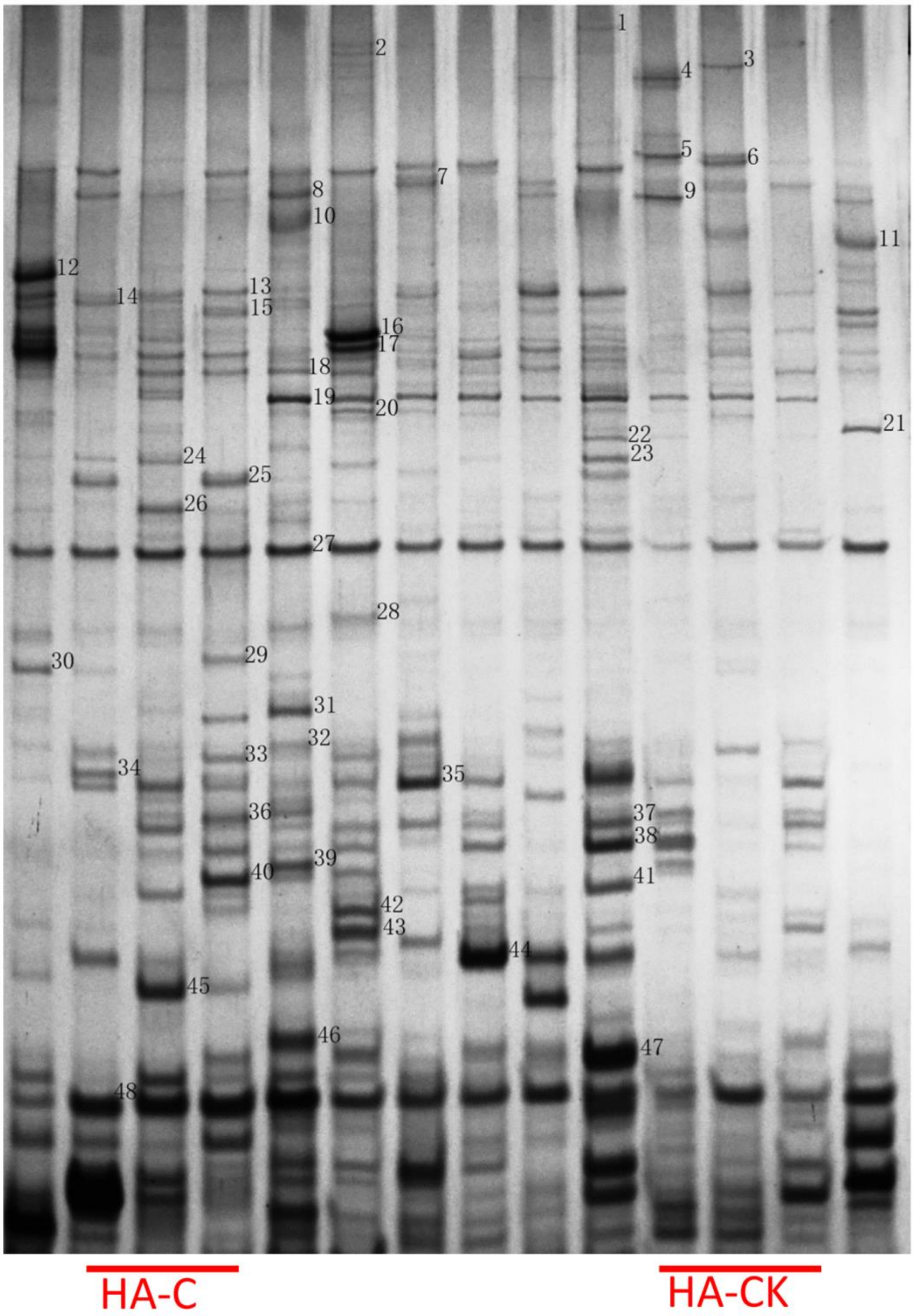

Figure 2 DGGE patterns of fungal ITS (F)
